# Idiopathic Left Subclavian Artery Dissection

**DOI:** 10.7759/cureus.12151

**Published:** 2020-12-18

**Authors:** Samantha Williams, Ana Pagan Jaramillo, Marie-Louise Posch, Sean-Patrick A Prince, Leonard Hamera

**Affiliations:** 1 Internal Medicine, HCA Healthcare/University of South Florida Morsani College of Medicine, GME Citrus Memorial Hospital, Inverness, USA

**Keywords:** sad, subclabian artery dissection, idiopathic sad, nontraumatic sad, spontaneous sad

## Abstract

We present a case of a 65-year-old male admitted for a small bowel obstruction who was incidentally found to have a left subclavian artery dissection on computed tomography angiogram (CTA) of the aorta. Non-traumatic subclavian artery dissection (SAD) is rare and only a few cases have been published in the literature. In this report, we review previously reported clinical presentations, subsequent treatments, and discuss factors that impact the choice between surgical vs conservative management.

## Introduction

Rarely, spontaneous or minimally traumatic subclavian artery dissections (SAD) have been reported, most likely due to its relative position in the body [1]. Lying just beneath the clavicles, injury to the subclavian artery is most likely to occur in the setting of trauma, vascular conditions, connective tissue abnormalities, or invasive procedures such as cardiac catheterization [1-3]. Our patient did not experience trauma but instead was admitted for a small bowel obstruction. An idiopathic SAD was incidentally noted through imaging to rule out other pathology.

## Case presentation

**Figure 1:**
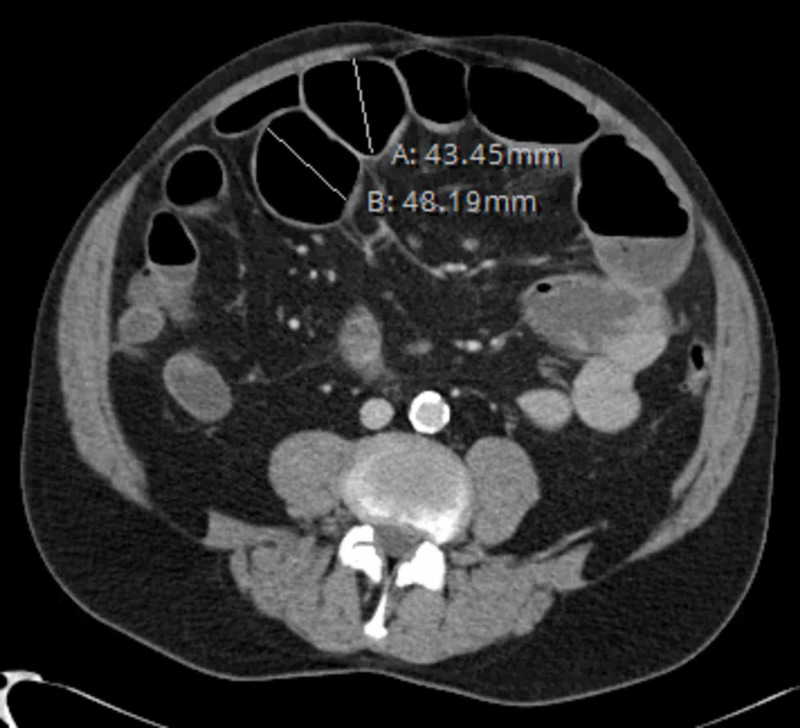
Computed tomography (CT) scan of the abdomen and pelvis showing a high grade small bowel obstruction with the small bowel lumen measuring up to 4.8 cm.

The patient is a 65-year-old male with a past medical history of hypertension who presented with a four-day history of abdominal pain, nausea, vomiting and diarrhea. Patient reported that the diarrhea subsided two days prior to presentation with no passage of stool or flatus since. On physical examination, initial vital signs were temperature 97.8°F, blood pressure 125/62 mmHg, and heart rate 95 beats per minute, and respiratory rate 18 breaths per minute. The patient's abdomen was distended and non-tender with hypoactive high pitched bowel sounds. Physical examination was otherwise unremarkable. Computed tomography (CT) scan of the abdomen and pelvis showed a high grade small bowel obstruction with the small bowel lumen measuring up to 4.8 cm. A nasogastric (NG) tube was placed for decompression of the stomach and abdominal X-ray was done to confirm the position (Figure 1).

On day 3 of admission, a rapid response was called because of tachycardia detected on telemetry. Patient was complaining of back pain and significant tenderness to palpation. Patient denied any blurry vision, headache, neck pain, chest pain or paresthesia and bilateral upper limb blood pressure readings revealed no major difference. Abdominal examination was unchanged from prior assessments.

**Figure 2:**
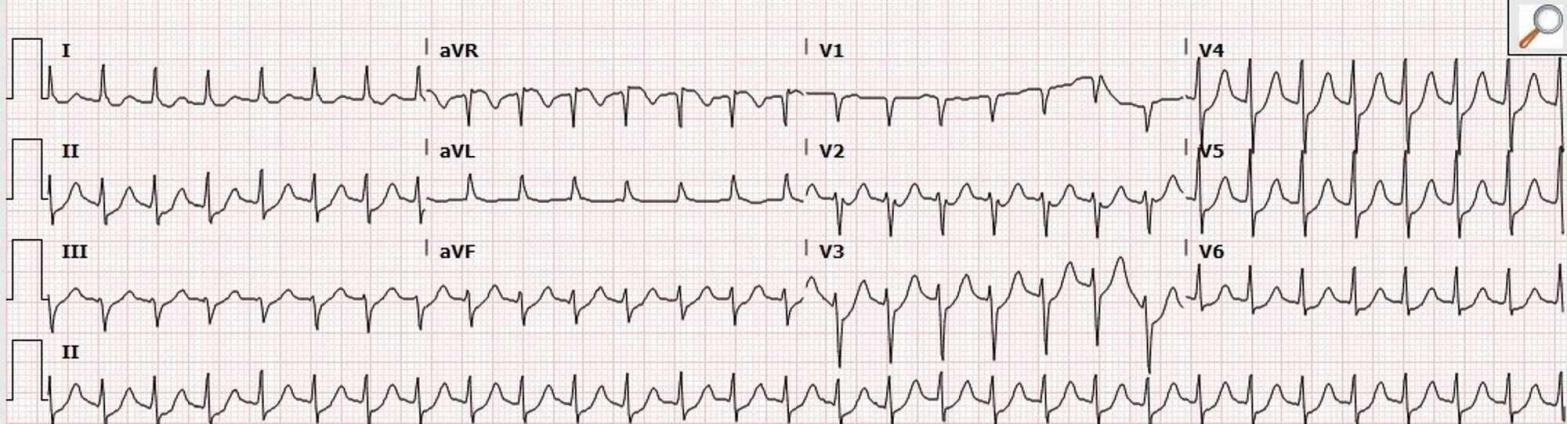
Electrocardiogram (ECG) displayed supraventricular tachycardia (SVT) with diffuse nonspecific ST depressions throughout.

**Figure 3:**
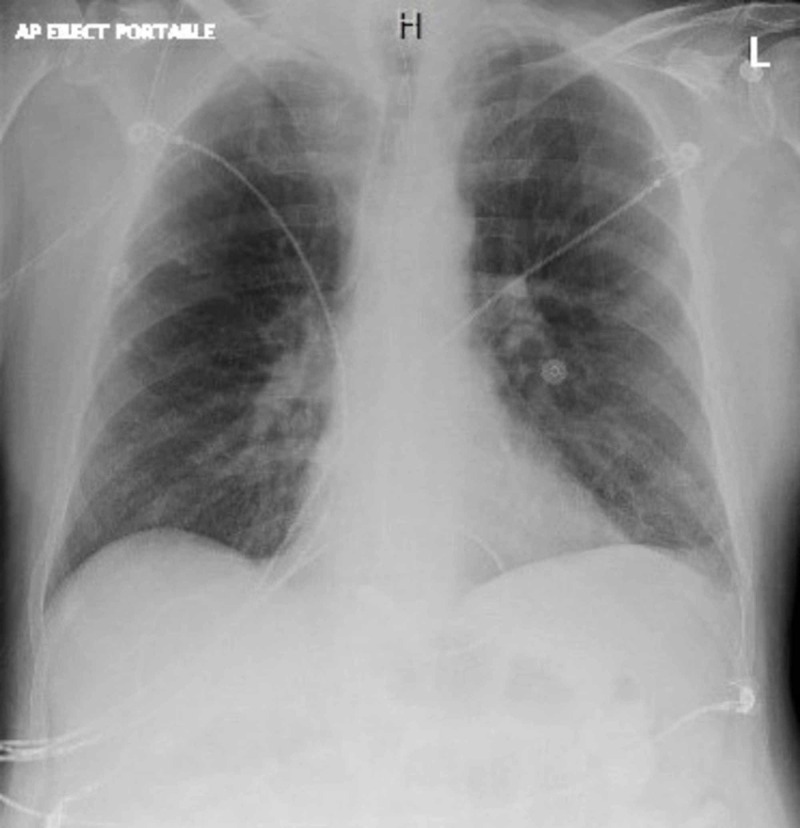
Portable chest X-ray negative for free air.

Vital signs showed elevated systolic blood pressures persistently greater than 160 mmHg and a heart rate over 170 beats per minute. Electrocardiogram (ECG) displayed supraventricular tachycardia (SVT) with diffuse nonspecific ST depressions throughout and a portable chest X-ray negative for free air (Figures 2-3).

**Figure 4:**
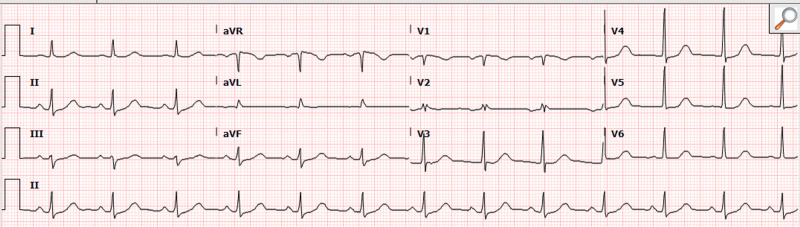
Repeat electrocardiogram (ECG) demonstrating resolution of supraventricular tachycardia (SVT) and normalization of ST segments.

Patient was treated with intravenous (IV) hydralazine and IV metoprolol 2.5 mg. Repeat ECG demonstrated resolution of SVT and normalization of the ST segments (Figure 4).

**Figure 5:**
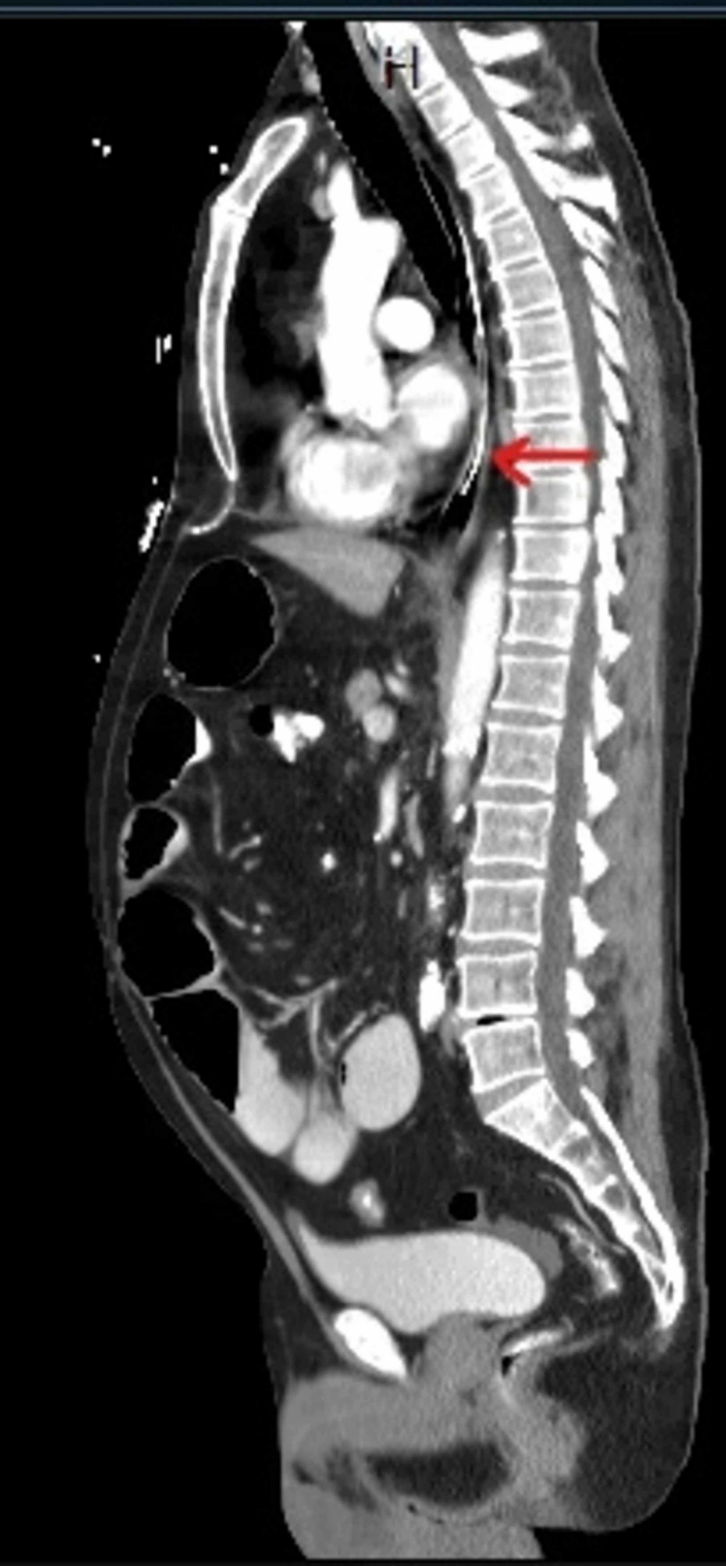
Tip of nasogastric tube malpositioned in the distal esophagus.

**Figure 6:**
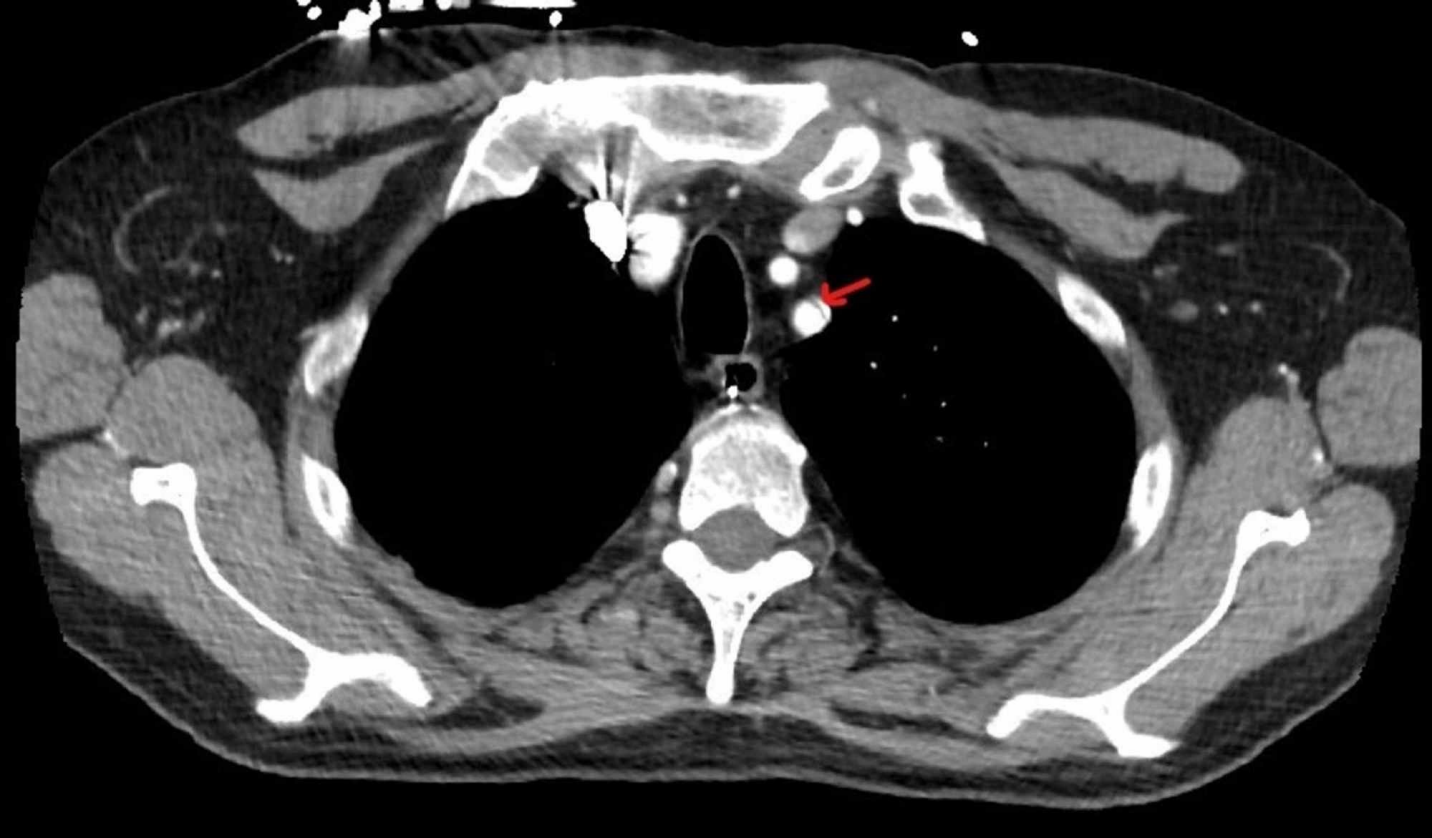
Left subclavian artery dissection (SAD) without involvement of the aorta or branch vessels on computed tomography angiogram (CTA) scan.

Due to persistent interscapular back pain and elevated blood pressure, a CT scan of the aorta was ordered to evaluate for an abdominal aortic aneurysm (AAA). Findings of the CT scan did not suggest evidence for a pulmonary embolism and/or AAA but rather revealed the tip of the NG tube malpositioned in the distal esophagus (Figure 5). Incidentally, CTA of the aorta also revealed a left SAD without involvement of the aorta or branch vessels (Figure 6).

**Figure 7:**
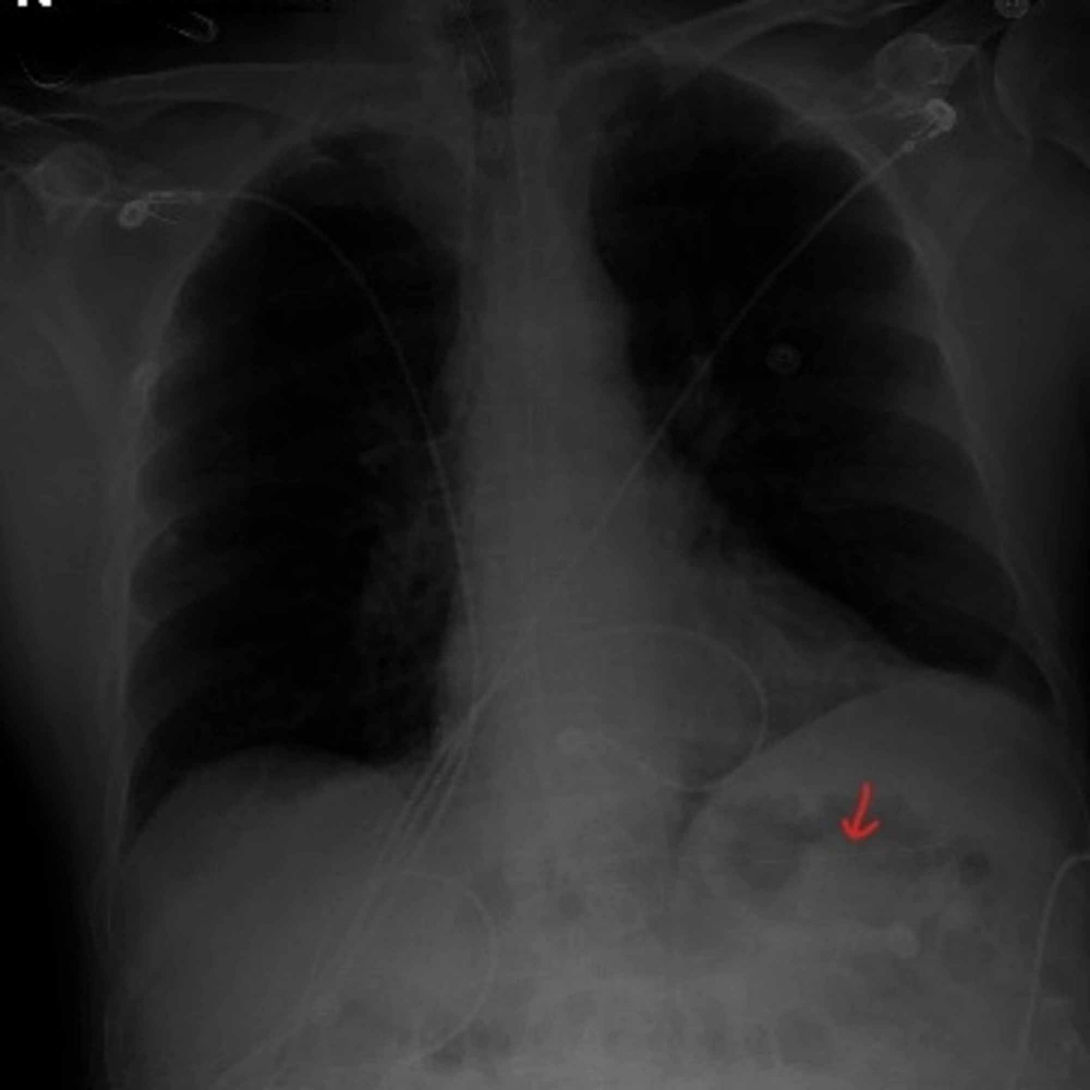
Chest X-ray confirming nasogastric tube within the stomach.

The NG tube was then advanced and correct positioning was confirmed on repeat imaging (Figure 7).

Vascular surgery was consulted to advise on appropriate management of subclavian artery dissection. Conservative management with patient’s outpatient medications, oral losartan and amlodipine, were eventually recommended as the patient’s symptoms resolved with NG tube repositioning. Patient was encouraged to follow up as an outpatient for repeat imaging to assess for extension of the dissection. Ultimately, the patient was taken to the operating room for laparoscopic adhesiolysis due to lack of resolution of small bowel obstruction. Post operatively, his condition improved and was discharged home to follow up for routine post-surgical assessments and monitoring of left SAD. At the time of reporting, patient failed to follow up as outpatient with vascular surgery.

## Discussion

SAD without a known cause is quite rare and not well understood [1]. Although spontaneous or minimally traumatic cases have been documented; most cases are secondary to trauma, connective tissue disease, arterial catheterization or aortic arch anomalies [4,5]. Usually, traumatic arterial dissections will involve the aortic isthmus and distal descending thoracic aorta but rarely the vessels above the aorta [5]. The precise pathogenesis of any arterial dissection is unclear but SAD has also been associated with migraines, pregnancy, and illicit substance use [6,7]. Overall, the incidence is higher in women in their 40s-50s with comorbid hypertension [5]. Our patient’s major risk factor for SAD was hypertension with no recent history of trauma, arterial catheterization or connective tissue disease.

Possible clinical presentations of SAD include back pain, thoracic pain, dizziness and arm paresthesia or other neurological symptoms mimicking that of a stroke [1,2,4-7]. Physical examination may detect different blood pressures between arms and/or signs of unilateral upper limb ischemia [5]. In one report, a patient presented with signs of ischemia affecting the cerebellum, spinal cord and cervical roots as a result of a SAD [2]. The back pain that was described in our case was most likely due to esophageal irritation secondary to NG tube mal-positioning. In the peer-reviewed literature, there has been one other reported case of asymptomatic or mildly symptomatic idiopathic SAD as in our patient [1].

Possible imaging modalities for evaluation of SAD include carotid Doppler ultrasound, although angiography and CTA remains the gold standard [5]. If neurologic deficits are present, magnetic resonance angiogram (MRA) has been found to be useful identifying lesions of the central nervous system. In our case, CTA clearly demonstrated a left SAD [3].

Although, possible complications such as intramural hemorrhage, false aneurysm, thrombosis or emboli to the head, neck or upper extremity have been reported, the overall prognosis of SAD is favorable [1,7]. The choice of management depends on the presence of any evolution of the false lumen, signs of ischemia or exacerbation of symptoms [1,5,6]. Uncomplicated cases have been shown to spontaneously resolve following conservative blood pressure therapy alone as seen in our case present [1,6]. Unfortunately, our case was lost to follow-up limiting further monitoring for resolution or progression. Heparin and antiplatelet therapy have also been used with good outcomes to manage SADs [1,4,7]. When indicated, in those with ischemic symptoms and/or significant comorbid conditions, endovascular stent-graft treatment has also been shown to have good outcomes [5,7]. Traditionally surgery and endovascular repair have been considered conventional treatments for symptomatic SAD; conservative therapy remains the suggested option for asymptomatic SAD when combined with careful follow-up and monitoring for progression [1,4,5,6].

## Conclusions

SAD is a rare finding and overall prognosis is usually favorable with good outcomes following conservative management with medical therapy alone, especially in asymptomatic patients. Although endovascular treatments have been found to result in good outcomes for those with symptoms; more research is necessary for standardization of treatment options. 
